# Nocturia in female patients: Current clinical features, treatment patterns and outcomes at a tertiary referral centre

**DOI:** 10.1080/2090598X.2019.1589792

**Published:** 2019-04-18

**Authors:** Siri Drangsholt, Benoit Peyronnet, Maria Arcila-Ruiz, Rachael D. Sussman, Ricardo Palmerola, Dominique R. Pape, Nirit Rosenblum, Victor W. Nitti, Benjamin M. Brucker

**Affiliations:** Department of Urology, New York University, New York, NY, USA

**Keywords:** Nocturia, female, desmopressin, voiding diary, urodynamic

## Abstract

**Objective**: To report the current clinical features, treatment patterns and outcomes of female patients who were seen at a tertiary referral centre with a primary diagnosis of nocturia, and to assess the predictive factors of therapeutic management failure.

**Patients and methods**: A retrospective chart review of all new female patients seen in a single-centre functional urology practice with the diagnosis of nocturia was performed. Up to three visits within a 12-month period from the time of presenting were reviewed. The primary endpoint was patient-reported improvement assessed at each follow-up visit and the change in the number of nocturia episodes.

**Results**: In all, 239 female patients were included for analysis. The prevalence of nocturnal polyuria, reduced bladder capacity, and global polyuria were 75%, 40.2%, and 18.1%, respectively. Within the first two visits, 72.7% of patients had started a treatment beyond behavioural therapies. Anticholinergics were the most commonly initiated treatment (47.2% of patients). At the latest considered visit, 80 patients reported improvement in nocturia (45.5%) and there was a mean – 0.8 decrease in the number of nocturia episodes from 4 to 3.2, which was statistically significant (*P* < 0.001). There was no statistically significant association between any of the bladder diary findings and treatment outcomes. A smaller number of nocturia episodes was the only predictive factor of therapeutic management failure in multivariate analysis (odds ratio 0.10; *P* = 0.01).

**Conclusions**: Whilst the prevalence of nocturnal polyuria in women with nocturia is high, the therapeutic management until 2016 seemed to rely mostly upon overactive bladder medications with a relatively low success rate.

**Abbreviations**: BD: bladder diary; BPS: bladder pain syndrome; ICD(−9)-(10): International Classifications of Disease (ninth revision) (10th revision); NPI: Nocturnal Polyuria Index; OAB: overactive bladder; OR: odd ratio; POP: pelvic organ prolapse

## Introduction

Nocturia is one of the most common and bothersome LUTS [1,2]. It has been viewed for long time as being a predominantly male symptom caused by BPH and gathered with other voiding and storage LUTS, under the umbrella term ‘prostatism’ [3,4]. However, over the past decade, several studies have shown that nocturia is as prevalent in women as it is in men and suggesting that this symptom may have been overlooked and underestimated in female patients for a long time [5,6]. This finding has assisted in the increasing recognition of the pathophysiological contributions of kidney and bladder dysfunctions, and numerous non-urological comorbidities (e.g. obstructive sleep apnoea, endocrine dysfunction, cardiovascular disease, etc.) to the symptom of nocturia in both men and women [6]. Although both genders share several pathophysiological mechanisms, the genesis of nocturia in male and female patients differs widely. The prostate can play a significant role in men, whilst gender-specific factors may be involved in women, such as hormonal status and dysfunctions, parity, history of gynaecological surgery, etc. [7,8]. As a result, one may assume that therapeutic patterns and response to treatment may differ according to gender for nocturia. The possibly slightly higher prevalence of overactive bladder (OAB) in women vs men [9] could also suggest a greater pathophysiological contribution of bladder storage issues likely to influence clinical features and therapeutic management. However, the literature on clinical presentations, treatment and outcomes of nocturia in female patients to confirm this hypothesis is scarce [7,8]. To date, the ICS does not distinguish male and female nocturia [10]. The primary objective of the present study was to report the current clinical features, treatment patterns and outcomes of female patients who were seen at a tertiary referral centre with a primary diagnosis of nocturia. The secondary objective was to assess the predictive factors of therapeutic management failure.

## Patients and methods

### Study design

A retrospective chart review of all new female patients seen in a single-centre functional urology practice with the diagnosis of nocturia was performed. Billing records were mined from 2010 to 2016, (a period after the initiation of electronic medical records) for patients with a primary diagnosis of nocturia, with an International Classifications of Disease ninth revision (ICD-9) code 788.43 and ICD-10 code R35.1. All patients were evaluated and treated by one of three Female Pelvic Medicine and Reconstructive Surgery (FPMRS)-certified urologists. The inclusion criterion was a primary diagnosis of nocturia. Patients were excluded if they were male, if they had undergone a treatment for bladder cancer, had a history of recurrent UTIs or had OAB predominant daytime symptoms. Up to three visits within a 12-month period from the time of presenting were reviewed.

### Initial and follow-up evaluations

The patients’ evaluation was not standardised and was individualised to each patient’s characteristics and clinical presentation. Physical examination, post-void residual and urine analysis were routinely performed. Other testing, such as bladder diary (BD) and/or urodynamics, was performed at the physician’s discretion. The number of nocturia episodes was self-reported by the patient during clinical interview, and recorded at each visit. The following patients’ characteristics were collected at the initial visit: age, body mass index, concomitant pelvic organ dysfunction [i.e. stress urinary incontinence, OAB, bladder pain syndrome (BPS), pelvic organ prolapse (POP), constipation]; comorbidities possibly contributing to nocturia (i.e. prior cardiac history, primary sleep disorder, diabetes, diuretic use, neurological condition); prior medications used for nocturia (i.e. α-blockers, β3 agonists, antimuscarinics, desmopressin); and prior genitourinary surgeries (i.e. midurethral slings, hysterectomy, POP repair). The duration from nocturia onset was also recorded during the first visit. Only complete records were included in the analysis of BD data. The following BD variables were collected: daytime voids, night-time voids, 24-h fluid intake and output, maximal voided volume, and 24-h Nocturnal Polyuria Index (NPI). Pathophysiological mechanisms based on BD interpretation were categorised as follows: nocturnal polyuria, reduced bladder capacity, and global polyuria [10]. BDs were analysed according to provider interpretation and retrospectively using ICS definitions. Nocturnal polyuria was defined as an NPI ≥ 0.33 for all ages for increased discrimination [11]. Reduced bladder capacity was subjectively defined as a maximum voided volume of < 250 mL.

### Therapeutic management

Behavioural treatments (fluid restriction, bladder irritant reduction, physical therapy, leg elevation, compressive stocking, etc.) were offered to all patients as first-line therapies, if they had not implemented these manoeuvres prior to presentation. Beyond this, conservative first-line therapeutic strategy was not standardised and left to the physician’s clinical judgement. Second-line recommended treatments were categorised as: α-blockers, anticholinergics, β3 agonists, desmopressin, vaginal oestrogens; and third-line OAB treatments (onabotulinum toxin A, percutaneous tibial nerve stimulation, sacral neuromodulation) and others. Only treatment given at the two first visits were considered and recorded for the present analysis.

### Outcomes of interest

The primary endpoint was patient reported improvement (vs no improvement) assessed at each follow-up visit. The other outcome of interest was the change in the number of nocturia episodes as self-estimated by the patient during clinical interview (i.e. not BD based). Therapeutic management failure was defined as the lack of any patient reported improvement at the latest considered visit (i.e. second or third).

### Statistical analysis

Means and standard deviations (SDs) were reported for continuous variables and proportions for nominal variables. The paired Student’s *t*-test was used to compare continuous variables across time. Predictive factors of therapeutic management failure were investigated using univariate and multivariate logistic regression analyses. Multivariable models included covariates with a *P* < 0.2 in univariable analysis. For continuous variables, odd ratios (ORs) were expressed as a range (per change in regressor over entire range). Statistical analyses were performed using JMP, version 14.0, software (SAS Institute Inc., Cary, NC, USA). All tests were two-sided with the significance level set at *P* < 0.05.

## Results

### Patients’ characteristics

After exclusion of 164 male patients, 239 female patients were included for analysis. The patients’ characteristics are summarised in Table 1. The mean age was 73.3 years, with a mean duration from nocturia onset of 2.4 years and a mean of 3.9 episodes of nocturia per night. In all, 91 patients had received at least one treatment for nocturia prior to their first visit to our site (38.1%), mostly anticholinergics. Most women had at least one concomitant pelvic floor dysfunction (64.4%), with 129 (54%) reporting daytime OAB symptoms. A majority of women (51.5%) had at least one comorbidity possibly contributing to their nocturia, with prior cardiac history and obesity being the most common (28.8% and 11.4%, respectively). A BD was suggested within the two first visits to 110 patients (46%), but was completed by only 72 patients (30.1%, completion rate = 65.5%). According to the BDs, the mean (SD) number of voids per 24 h was 11.1 (4), the NPI was 41.2 (16.8)%, and the maximum voided volume was 364 (222.1) mL. The prevalence of nocturnal polyuria, reduced bladder capacity, and global polyuria were 75%, 40.2%, and 18.1%, respectively. Excessive fluid intake was identified as the causative factor of the vast majority of global polyuria cases (76.9%).

**Table 1.** Patients’ characteristics.

Variable	Value
Total number of female patients with nocturia	239
Mean (SD):	
Age, years	73.3 (15.1)
Duration from nocturia onset, years	2.4 (4.2)
Number of nocturia episodes	3.9 (1.7)
Body mass index, kg/m^2^	24.8 (5.3)
*N* (%):	
History of genitourinary surgery	64 (26.9)
Midurethral sling	13 (5.6)
POP repair	25 (10.7)
Hysterectomy	38 (16.2)
History of prior medications for nocturia	91 (38.1)
α-blockers	5 (2.1)
Anticholinergics	69 (28.9)
β3-agonists	7 (2.9)
Desmopressin	6 (2.5)
Concomitant pelvic floor dysfunction	154 (64.4)
OAB	129 (54)
BPS	8 (3.4)
POP	24 (10.1)
Stress urinary incontinence	24 (10.1)
Constipation	13 (5.5)
Comorbidities possibly contributing to nocturia	123 (51.5)
Past cardiac history	68 (28.8)
Primary sleep disorder	13 (5.5)
Obesity	27 (11.4)
Diuretic use	6 (2.5)
Diabetes mellitus	9 (3.8)
Neurological condition	6 (2.5)
Urodynamic testing performed within the two first visits	35 (14.6)
BD	
Suggested	110 (46)
Completed	67 (28)

### Treatment patterns and outcomes

**Figure 1.**
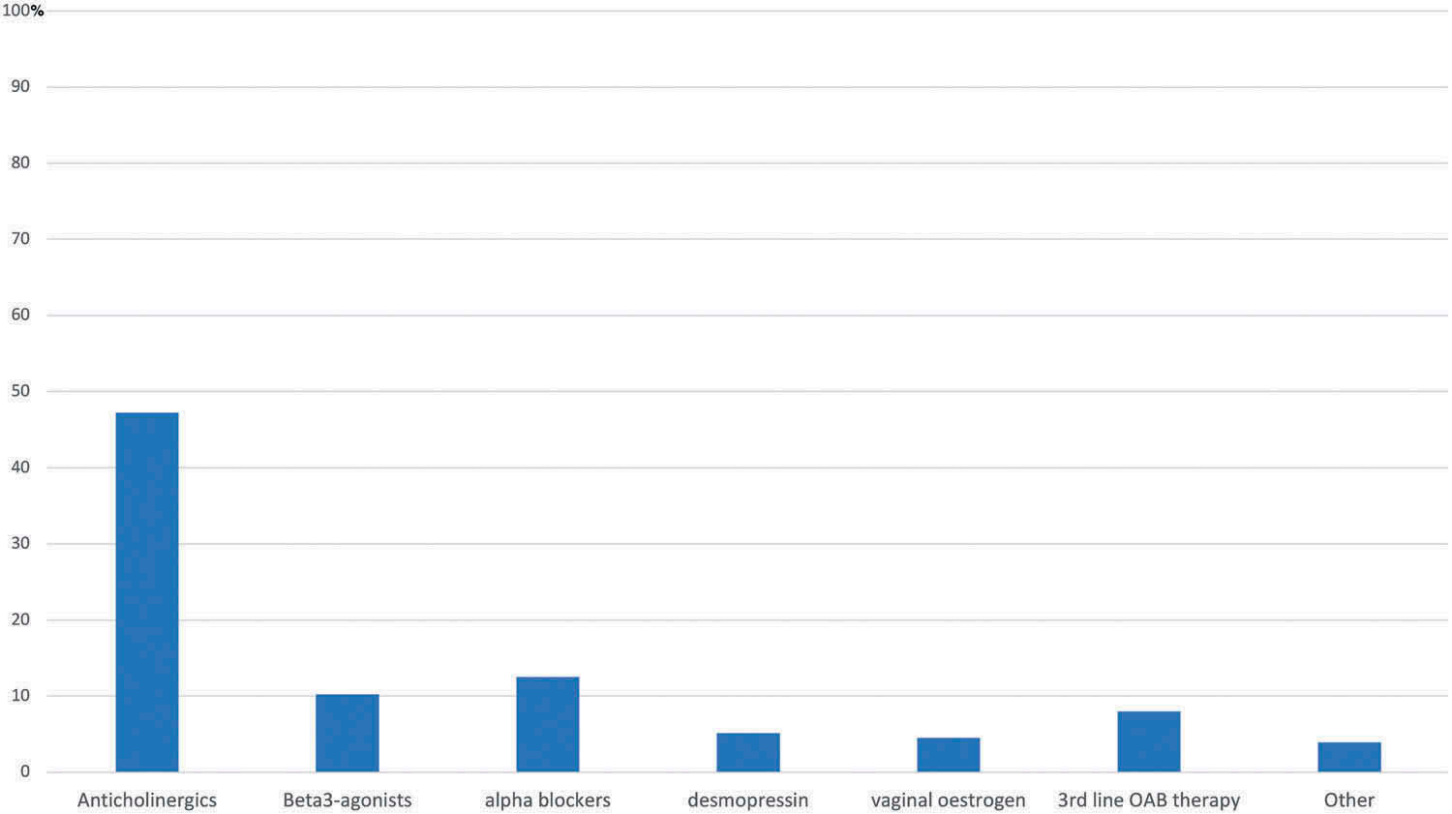
Medical treatments initiated for nocturia within the two first visits.

Of the entire cohort, 176 patients (83.6%) returned for a second visit and were eligible for treatment analysis. Only 112 patients (46.9%) were followed-up until a third visit. Within the first two visits, 128 of 176 patients (72.7%) had started a treatment beyond behavioural therapies. The distribution of these treatments is shown in Figure 1. The most common treatments initiated were anticholinergics (83 patients, 47.2%), α-blockers (22, 12.5%), and β3 agonists (18, 10.2%). Overall, 93 patients (52.8%) were treated with OAB medications (anticholinergics and/or β3 agonists). Only nine patients (5.1%) were started on desmopressin.

At the latest considered visit (i.e. second or third), 80 patients (45.5%) reported improvement of nocturia and there was a median decrease from 4 to 3 nocturic episodes, and a mean (SD) – 0.8 (1.5) decrease of episodes from 4 to 3.2, which was statistically significant (*P* < 0.001).

### Predictive factors of therapeutic management failure

In univariate analysis, neither the completion of a BD (OR 0.98; *P* = 0.94), the use of anticholinergics (OR 0.86; *P* = 0.64), of α-blockers (OR 0.82; *P* = 0.70), of β3 agonists (OR 1.09; *P* = 0.87), or of desmopressin (OR 0.83; *P* = 0.80) was associated with therapeutic management failure. The number of nocturia episodes at baseline was the only predictive factor of therapeutic management failure (OR 0.09; *P* = 0.005). In multivariate analysis adjusting for the coexistence of OAB and obesity, fewer nocturia episodes at baseline remained the only significant predictive factor of therapeutic management failure (OR 0.10; *P* = 0.01). In a subgroup analysis focusing on patients who completed a BD, there was no statistically significant association between any of the BD findings (i.e. maximum voided volume, number of voids per day, NPI, nocturnal polyuria, global polyuria) and treatment outcomes.

## Discussion

Nocturia negatively impacts quality of life and contributes to impaired sleep and overall health [12]. It is the leading cause of sleep disturbance in patients aged > 50 years, places elderly patients at risk of falling, and is associated with increased mortality [12]. Nocturia has been found to equally affect men and women [5,6]. However, the pathophysiology of nocturia, and inherently the treatment patterns and outcomes, may differ widely between male and female patients [7,8]. Possibly because nocturia has for a long time been regarded as a prostatic symptom [3,4], the specificities of nocturia in female patients have rarely been studied [7,8]. In the present study, we found that nocturic women are an elderly and significantly comorbid patient population. Whilst nocturnal polyuria appeared to be the main pathophysiological mechanism, women were mostly offered treatments targeting bladder dysfunction with relatively poor outcomes.

The pathophysiological mechanisms underlying nocturia fall into three main categories: nocturnal polyuria, global polyuria, and reduced bladder capacity; the latter being often caused by bladder storage problems, such as OAB or BPS [10]. Interestingly, despite OAB, and even more BPS, being known to be slightly more prevalent in female than in male patients [9,13], nocturnal polyuria was largely predominant over reduced bladder capacity in our present female nocturic population. However, the therapeutic management largely relied on OAB medications targeting bladder storage problems rather than night-time urine overproduction. Whilst there was a statistically significant improvement with – 0.8 nocturia episodes at the follow-up visit, this may not be clinically significant. The impact of one less nightly void may depend on the baseline nocturia number and bother to the patient. One may hypothesise that this mismatch between pathophysiological mechanisms and treatment patterns could have contributed to the relatively low improvement we observed in this population of female patients with nocturia.

Medication directed towards overproduction of urine could be regarded as a promising option for improving the outcomes of nocturia therapeutic management. Desmopressin, an analogue of arginine vasopressin, treats excessive urine production by increasing fluid reabsorption by the kidney leading to more concentrated urine production. Despite having been available for years, the formulations of desmopressin on the market over the study period were used in only 5.1% of the women, probably because of a lack of regulatory approval for nocturia, unpredictable pharmokenetics, and fear of associated side-effects [14]. The introduction of recently approved desmopressin formulations may change nocturia treatment patterns [15,16]. Newly engineered medications have highly predicable pharmokenetics profile and thus lower rates of hyponatraemia, with the same effect as available desmopressin tablets [15,16,17]. Further ‘real-life’ studies will be needed to determine if the recent approval of these new therapies will translate into a larger use of desmopressin containing medications, and ultimately in enhanced pharmacological benefits in female patients with nocturia.

BDs remain the best tool to narrow the pathophysiological mechanisms for nocturia into nocturnal polyuria, global polyuria, diminished bladder capacity, or combined mechanisms. However, BDs can be labour intensive and result in variable compliance [18]. In the present series, a BD was prescribed to only 46% of patients and completed by only 30.1% of the entire cohort. Paradoxically, the high volume of the providers involved may explain this underutilisation of BDs, these urologists relying mostly upon thorough medical, medication, voiding and fluid consumption history, and upon their clinical expertise to focus therapy. The lack of approved pharmacological treatment targeting nocturnal polyuria at the time of the study period could also have contributed to a less aggressive screening of this mechanism. The completion of a BD was not associated with therapeutic management outcomes in our present series. This finding might be different in the newer era of USA Food and Drug Administration (FDA)-approved treatment for nocturnal polyuria. Future research is needed to delineate if anti-diuretic therapy should be restricted to those with only BD-confirmed nocturnal polyuria. How we used BDs in the future may change as there are differences in treatment patterns and perhaps more data on how the results impact treatment choices.

The present study has several limitations that should be acknowledged. First, it has all the biases inherent to its retrospective single-centre design. The relatively high rate of patients lost to follow-up (only 46.9% of patients attended at least three visits) is an important shortcoming and may have overestimated the disappointing outcomes of treatment prescribed at the first encounter. This may have been one of the reasons patients did not return for follow-up visits. Moreover, only three visits were considered and analysed in the present study, whilst one may argue that therapeutic management changes and outcomes may have evolved beyond the third visit. Behavioural therapies were not standardised across patients and providers, and this factor, likely to influence the results, could not be controlled for. The assessment of comorbidities may have been underestimated, as these were obtained from the clinical visits and from prior documentation in the electronic medical record, which may or may not have been input from a primary care physician or geriatrician. Most patients do not require a referral from primary care, thus a patients history may have little collateral to help establish risk factors, which may have helped tailor treatment. Finally, there was no validated outcomes or questionnaires used. The patient-reported improvement in nocturia is not a standardised assessment and is subject to several biases (e.g. influence of the provider). The assessment of nocturia as reported per patient during clinical interview has also been largely criticised by being poorly reliable and commonly overestimating the number of nocturia episodes [19].

## Conclusion

Women presenting at a tertiary referral centre with nocturia appeared to be an elderly and significantly comorbid population. Whilst the prevalence of nocturnal polyuria in women with nocturia is high, the therapeutic management until 2016 seemed to rely mostly upon OAB medications. The relatively low rate of patient-reported improvement could suggest that targeting nocturnal polyuria might be of interest in order to address the nocturic women’s expectation. Re-assessment of treatment patterns and outcomes after the recent approval by regulatory authorities of newer desmopressin formulations for nocturia would be needed to confirm this hypothesis.
